# Surveyed veterinary students in Australia find ChatGPT practical and relevant while expressing no concern about artificial intelligence replacing veterinarians

**DOI:** 10.1002/vro2.80

**Published:** 2024-06-09

**Authors:** Kate A. Worthing, Madeleine Roberts, Jan Šlapeta

**Affiliations:** ^1^ Sydney School of Veterinary Science Faculty of Science The University of Sydney Sydney New South Wales Australia; ^2^ Sydney Infectious Diseases Institute The University of Sydney Sydney New South Wales Australia

## Abstract

**Background:**

Chat Generative Pre‐trained Transformer (ChatGPT) is a freely available online artificial intelligence (AI) program capable of understanding and generating human‐like language. This study assessed veterinary students’ perceptions about ChatGPT in education and practice. It compared perceptions about ChatGPT between students who had completed a critical analysis task and those who had not.

**Methods:**

This cross‐sectional study surveyed 498 Doctor of Veterinary Medicine (DVM) students at The University of Sydney, Australia. Second‐year DVM students researched a veterinary pathogen and then completed a critical analysis of ChatGPT (version 3.5) output for the same pathogen. A survey based on the Technology Acceptance Model was then delivered to all DVM students from all years of the programme, collecting data using Likert‐style, categorical and free‐text items.

**Results:**

Over 75% of the 100 respondents reported having used ChatGPT. The students found ChatGPT's output relevant and practical for their use but perceived it as inaccurate. They perceived ChatGPT output to be more useful for veterinary students than for pet owners or veterinarians. Those who had completed the critical analysis assignment had a more positive view of ChatGPT's practicality for veterinary students but noted its authoritative tone even when delivering inaccurate information. Over 50% of the students agreed that information about tools such as ChatGPT should be included in the veterinary curriculum. Students agreed that veterinarians should embrace AI but disagreed that AI would eventually replace the need for veterinarians.

**Conclusions:**

A critical appraisal of outputs from AI tools such as ChatGPT may help prepare future veterinarians for the effective use of these tools.

## INTRODUCTION

The rise of artificial intelligence (AI) has been met with both excitement and trepidation among those working in veterinary medicine and education. The potential uses of AI in veterinary medicine include interpreting radiographs,^1^, ^2^ prognosticating cases^3^ and editing scientific papers.^4^, ^5^ Access to AI has been democratised with the arrival of online large language models (LLMs), such as Chat Generative Pre‐trained Transformer (ChatGPT). These models are capable of understanding and generating natural human language^6^; they provide freely accessible information to veterinarians, veterinary students and pet owners alike. Concerns have been raised in the education sector that the proliferation of LLMs, such as ChatGPT, could lead to widespread dissemination of misinformation and breaches in academic integrity.^7^ Such concerns arise from reports that ChatGPT can pass examinations designed for veterinary^8^ and dentistry students^9^ and produce essays with fake references.^4^ These findings highlight both the potential utility of AI, such as ChatGPT, for veterinary practitioners and the danger of using ChatGPT as a source of misinformation for veterinary students.

To design effective educational programmes with and about AI in veterinary curricula, we must understand current perceptions of veterinary students about AI and ChatGPT and their current or expected usage patterns of these tools. The Technology Acceptance Model (TAM) is a theoretical framework that can help gauge the likelihood that an individual will engage with a new technology.^10^ TAM posits that perceived usefulness and perceived ease of use are the key determinants of an individual's acceptance of technology. In the context of AI in veterinary medicine, perceived accuracy and perceived relevance of a technology to an individual's job can be considered proxies for perceived usefulness^11^, ^12^ and perceived practicality can be a proxy for ease of use.^13^ To the best of the authors’ knowledge, no studies have examined veterinary students’ perceptions of the perceived usefulness (accuracy and relevance) and perceived ease of use (practicality) of AI models such as ChatGPT.

With this in mind, we designed an assessment task that required second‐year Doctor of Veterinary Medicine (DVM2) students to critically analyse ChatGPT output about a veterinary pathogen with which they were familiar. Then, using a modified TAM framework, we delivered a post‐assessment survey to all students in the DVM course. We aimed to answer the following questions. (1) What are veterinary students’ perceptions about the accuracy, relevance and practicality of ChatGPT output for students, veterinarians and pet owners? (2) How do veterinary students perceive the impact of AI on veterinary medicine? (3) Do perceptions about ChatGPT differ between students who have completed a critical analysis task and those who have not?

## METHODS

### Research design and sample population

This prospective cross‐sectional study involved the distribution of an online survey to DVM students at The University of Sydney, Australia. The survey was delivered to all students (*n* = 498) in all years of the DVM programme, which is a contemporary 4‐year post‐graduate programme. The DVM curriculum is broadly aligned with Bloom's three domains of learning,^14^ including addressing cognitive learning through lectures and tutorials, psychomotor learning through practicals and clinical skills classes, and clinical placements addressing all learning domains, including affective learning. The curriculum is body system‐based (e.g., musculoskeletal system) rather than discipline‐based (e.g., pathology), with instructors and students encouraged to approach teaching and learning in an integrated multidisciplinary fashion. Around 45% of students in the DVM programme are from countries other than Australia, including the United States and China. By mid‐2023, all students in the DVM programme had access to basic information about ChatGPT. The default policy at The University of Sydney was that use of AI was not permitted in any assignment unless it was explicitly approved by an instructor. Second‐year Doctor of Veterinary Medicine (DVM2) students had ChatGPT (version 3.5) mentioned formally in lectures, where various outputs and prompts were shown to students, including the existence of fake citations in ChatGPT output. DVM2 students also undertook a critical analysis task involving ChatGPT as part of their ‘visual learning tool (VLT)’ assignment, as outlined below. If ChatGPT was covered in other years of the DVM curriculum, it was provided on an ad hoc basis by individual instructors. However, ChatGPT was available online for 4 months before the assessment task was given to DVM2 students and 6 months before the survey was delivered to all students.

**FIGURE 1:**
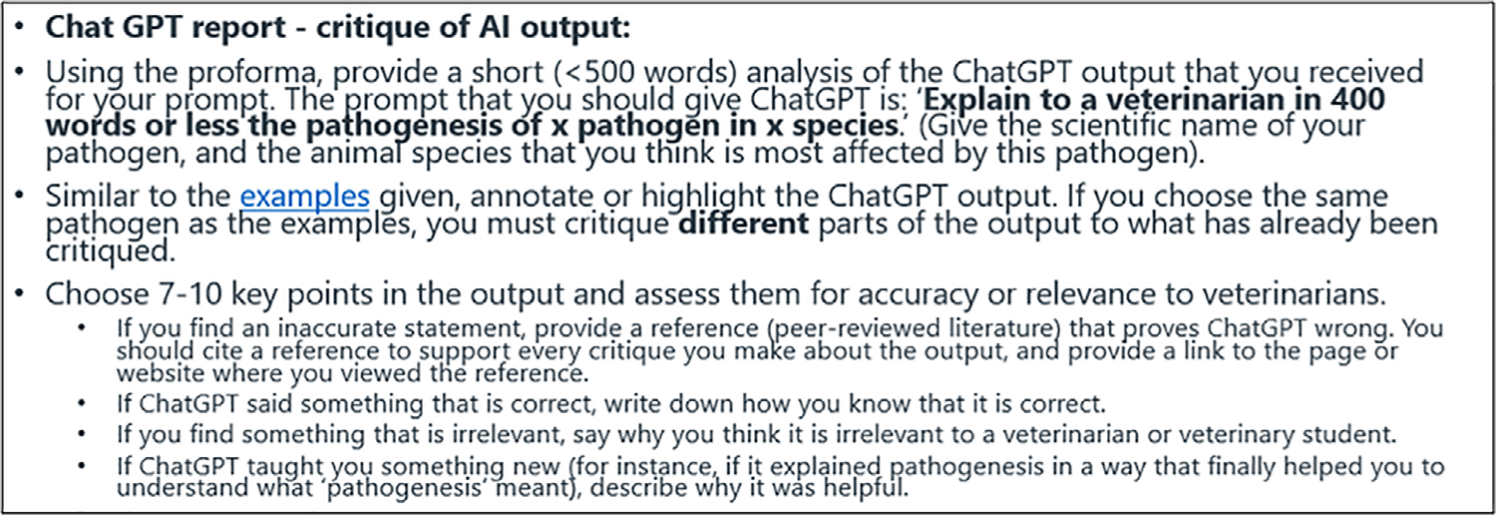
Instructions given to second‐year Doctor of Veterinary Medicine students after they had completed their own research about a veterinary pathogen and were asked to critique artificial intelligence (AI) output about the same pathogen. ChatGPT, Chat Generative Pre‐trained Transformer.

The survey was delivered 1 month after DVM2 students completed a critical analysis task. This task was a summatively assessed group assignment, which required DVM2 students to research a common veterinary pathogen and create VLT that could be used by veterinary students or veterinarians (poster, video, story book or infographic). Once student groups had researched and developed their VLT, they then had to critically appraise the ChatGPT (version 3.5) output generated in response to the prompt: ‘Explain to a veterinarian in 400 words or less the pathogenesis of *x* pathogen in *x* animal species’. The exact instructions given to the students about the AI critique assignment are shown in Figure 1.

### Measures

The 15 survey items included categorical items with ordinal or nominal responses, Likert‐style items with five‐point (ordinal, bipolar) response anchors^15^ and free‐text items. Items related to previous knowledge and use of AI tools such as ChatGPT, year of DVM enrolment, perceptions about the impact of AI on veterinary medicine and items based on modified key constructs of the TAM were used.^11^ For a detailed description of the survey items, see Appendix S1. The survey can be found in Appendix S2.

### Data collection procedure

Approval for the survey was obtained through the Human Ethics Committee at The University of Sydney (2023/346). The survey was built and delivered using Qualtrics. A link to the survey was distributed via an online announcement system (Student Relationship Engagement System, University of Sydney and University of Melbourne) to all DVM students at The University of Sydney. The survey was available to students from 27 July 2023 to 27 September 2023 (the break between semesters 1 and 2).

### Data preparation

The data were exported as a CSV file from Qualtrics into Microsoft Excel (version 16.0) to assess missingness of the data. Completion of questions was voluntary and, as such, questions could be skipped by respondents. Some survey items were only given to participants if they answered ‘yes’ to ‘Has ChatGPT been discussed in the veterinary education you have received?’. These data points were missing not at random (MNAR). Missing not at random data were removed by listwise deletion for analyses and the denominator was adjusted to the number of respondents who had asked all questions in an analysis. Besides the anticipated MNAR data points, to check for other types of missing data, the data were viewed in Excel to determine if missing data were missing completely at random, missing at random (MAR) or MNAR. Incomplete surveys with MAR data were still included in the descriptive statistics if the respondent had completed more than 20% of the survey because their missingness related to question order in the survey and not to the question itself (earlier questions were answered by more participants).^16^ When comparing responses of one question to another, cases with missing data for one or more questions were excluded in a listwise manner before statistical analysis. Comparative analyses were conducted using the number of responses for each question as the denominator.

### Data analysis

Descriptive statistics were performed in GraphPad Prism (version 10.1.1), while the remaining analyses were performed in Qualtrics. For reporting descriptive statistics of Likert‐style items, ‘somewhat agree’ and ‘strongly agree’ were reported as ‘agree’, while ‘somewhat disagree’ and ‘strongly disagree’ were reported as ‘disagree’. For statistical analyses involving Likert‐style items, each response was automatically assigned a score in Qualtrics from 1 to 5 (1 = strongly disagree, 2 = somewhat disagree, 3 = neither agree nor disagree, 4 = somewhat agree, 5 = strongly agree). Responses were reported as ‘agreed’ if the median response score from a cohort was 4 or more and ‘disagreed’ if the median response score was 3 or less. Figures were created by exporting data into Microsoft Excel and creating pivot tables to depict the percentage of responses to each Likert‐style item.

Ranked analysis of variance (ANOVA) (Kruskal‒Wallis test) was used to determine if there were differences in item responses based on cohort of study (DVM year 1, 2, 3 or 4). When a difference between groups approached significance (*p* < 0.1) according to ANOVA, a rank‐transformed Welch's *t*‐test for non‐parametric data was used to determine if differences existed between responses from DVM2 and other cohorts.^17^ Ranked ANOVA was also used to determine if there were significant differences between previous use of ChatGPT (used before, heard of but never used) and Likert item responses. The chi‐square test was used to determine if differences in prior use of ChatGPT existed between proportions of students from different cohorts. To determine the reliability of our Likert‐style TAM matrix items in measuring technology acceptance as a construct, we used a pre‐composed R script in Qualtrics to measure the McDonald's Omega coefficient.^18^ A high value of McDonald's Omega (close to 1) indicates that the items on a Likert scale or question matrix measure the same underlying construct; thus, the scale has good internal consistency.

Content analysis was undertaken on the free‐text responses within Qualtrics. Keywords were searched for terms such as ‘inaccurate’, ‘accurate’ and ‘change’; answers with these terms were grouped into categories using the ‘topics’ tool. The proportions of responses that fell into each comment category were then calculated. Interesting and well‐articulated comments that represented general themes were chosen for inclusion verbatim in the manuscript. A *p*‐value of less than 0.05 was considered statistically significant. All *p*‐values less than 0.0001 were reported as <0.0001.

## RESULTS

A total of 100 students responded to the survey, representing a response rate of 20%. One hundred and forty‐three DVM2 students completed the critical analysis assessment task. Of those, 43 (30%) participated in the survey. Three respondents were excluded because they did not answer anything beyond the first question of the survey. Data from 97 respondents were subsequently included in the analyses. Complete data were available for 93 respondents. The remaining four respondents had completed between 21% and 74% of the survey. Twenty‐six percent of respondents came from DVM1 (*n* = 25), 41% from DVM2 (*n* = 40), 16.5% from DVM3 (*n* = 16) and 16.5% from DVM4 (*n* = 16).

### Previous experience with ChatGPT

All respondents indicated that they had heard of ChatGPT before and 77.3% indicated that they had used it before. DVM2 students were more likely to have used ChatGPT than students in other years (92.5% vs. 66.7%, *p* = 0.003) (Table 1). More DVM2 students had ChatGPT discussed in their veterinary education than students in other years (100% vs. 53%, *p* < 0.0007). Fourteen of 16 (87.5%) DVM3 students responded that ChatGPT had been discussed in their education, attributable to a research and enquiry assignment where they were instructed to acknowledge whether or not that they had used ChatGPT.

**TABLE 1:** Doctor of Veterinary Medicine (DVM) students’ cohort and previous experience with Chat Generative Pre‐trained Transformer (ChatGPT).

	DVM1	DVM2	DVM3	DVM4	Total
What is your prior experience with ChatGPT?					
I've heard of ChatGPT but never used it before	11/25 (44%)	3/40 (7.5%)	2/16 (12.5%)	6/16 (37.5%)	75/97 (77.3%)
I've used ChatGPT	4/25 (56%)	37/40 (92.5%)	14/16 (87.5%)	10/16 (62.5%)	22/97 (22.7%)
Has ChatGPT been discussed in the veterinary education you have received?					
Yes	11/25 (44%)	39/39 (100%)	14/16 (87.5%)	5/16 (31.3%)	69/96 (71.9%)
No	14/25 (56%)	0/39 (0%)	2/16 (22.5%)	11/16 (68.8%)	27/96 (28.1%)

Responses to survey questions about ChatGPT in veterinary education are shown in Table 2. Among the 69 students who said that they had had ChatGPT as discussed in their veterinary education, there was general agreement with the statement ‘Prior to the discussion about ChatGPT in my veterinary education, I was already familiar with the strengths and weaknesses of AI tools such as ChatGPT’ (median Likert score = 4; Table 2). Students generally agreed with the statement ‘Following the discussion about ChatGPT in my veterinary education, I was more familiar with the strengths and weaknesses of AI tools such as ChatGPT’ (median score = 4), but there was a significant difference in responses depending on cohort (*p* = 0.001). DVM2 students had a higher agreement score (median = 4) with this statement than students in other years (median = 3, *p* = 0.001).

**TABLE 2:** Doctor of Veterinary Medicine (DVM) students’ cohort and agreement with statements about discussion of Chat Generative Pre‐trained Transformer (ChatGPT) in the curriculum.

	DVM1 (*n* = 11)	DVM2 (*n* = 39)	DVM3 (*n* = 14)	DVM4 (*n* = 5)	Total (*n* = 69)
Prior to the discussion about ChatGPT in my veterinary education, I was already familiar with the strengths and weaknesses of artificial intelligence tools such as ChatGPT.					
Median score	4.0	3.0	4.0	4.0	4.0
Following the discussion about ChatGPT in my veterinary education, I was more familiar with the strengths and weaknesses of artificial intelligence tools such as ChatGPT.					
Median score	2.0	4.0	3.5	4.0	4.0

*Note*: Response anchors—1 = strongly disagree, 2 = somewhat disagree, 3 = neither agree nor disagree, 4 = somewhat agree and 5 = strongly agree.

### Perceptions about the accuracy, relevance and practicality of ChatGPT

**FIGURE 2:**
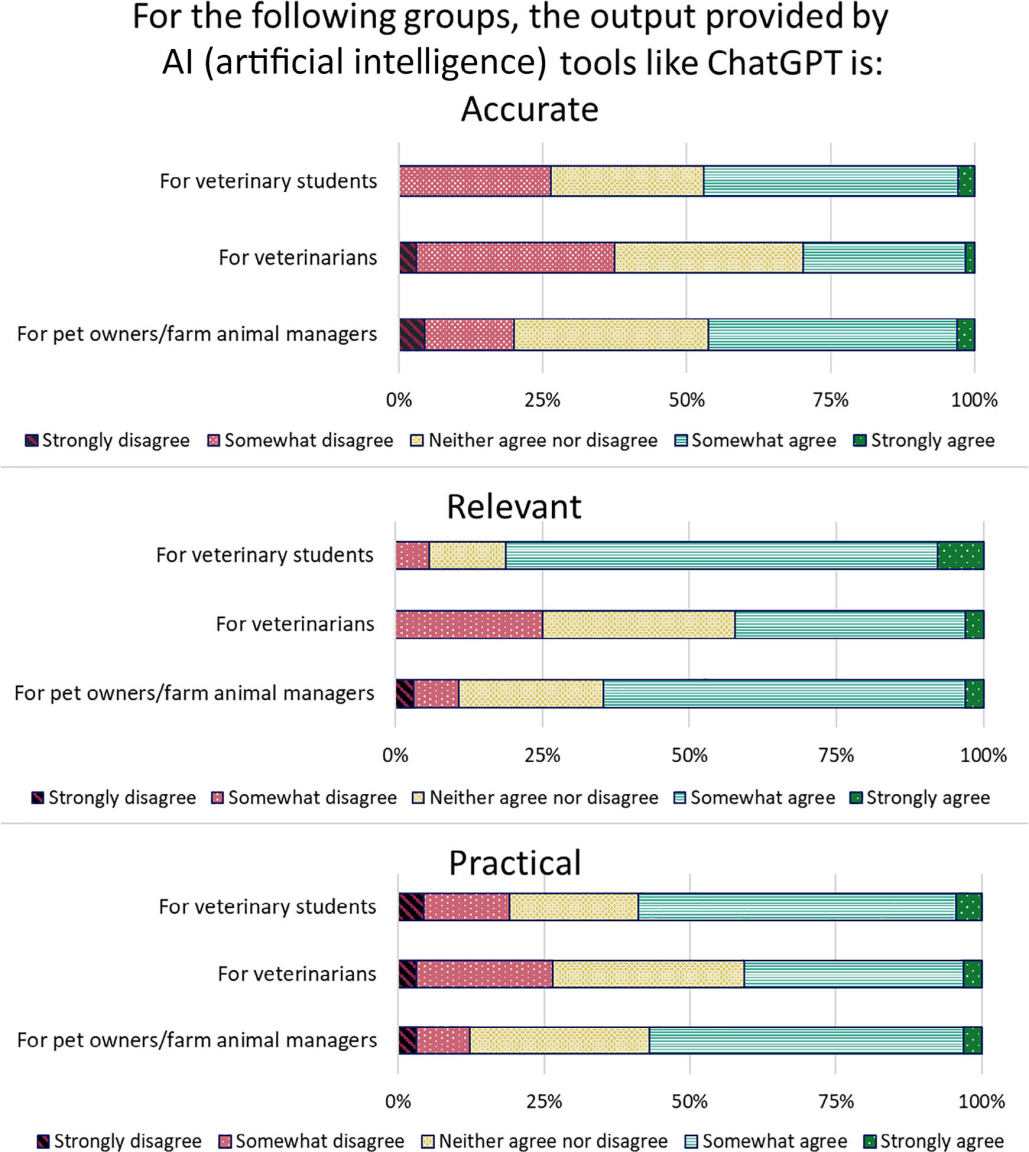
Doctor of Veterinary Medicine (DVM) students’ perceptions about the accuracy, relevance and practicality of Chat Generative Pre‐trained Transformer (ChatGPT) output. *N* = 68 DVM students from years 1 to 4 at the Sydney School of Veterinary Science.

Figure 2 illustrates the frequency of responses to the TAM items regarding DVM students' perceptions about accuracy and relevance (perceived usefulness) and practicality (perceived ease of use). There was good internal consistency for responses to ‘For pet owners/farm animal managers, the output provided by AI tools such as ChatGPT is accurate/relevant/practical’ and ‘For veterinarians, the output provided by AI tools such as ChatGPT is accurate/relevant/practical’ (McDonald's Omega = 0.84 and 0.83, respectively), meaning that when students tended to agree or disagree with one statement within an item, they would agree or disagree, respectively, with other statements in that item. However, the internal consistency was poor for ‘For veterinary students, the output provided by AI tools such as ChatGPT is accurate/relevant/practical’ (0.54). Consequently, all item responses in the TAM matrix were analysed separately rather than being aggregated into a TAM factor.^16^

Responses to TAM matrix questions were not significantly different across the DVM years of enrolment, so responses across cohorts were combined (Figure 2). The statement that had the highest agreement was ‘For veterinary students, the output provided by ChatGPT is relevant’ (82.9%). Responses to the statement ‘The output provided by AI tools such as ChatGPT is relevant’ were significantly different between ‘For veterinary students (median = 4)’, ‘For pet owners/farm managers (median = 4)’ and ‘For veterinarians’ (median = 3) (*p* < 0.0001). In general, more students agreed that ChatGPT was accurate (47%), relevant (82.9%) and practical (58.8%) ‘For veterinary students’ than ‘For pet owners’ (46.2%, 64.6% and 56.9%, respectively) and ‘For veterinarians’ (29.7%, 42.4% and 40.6%, respectively), but these differences were only significant for responses to ‘relevant’ (*p* < 0.0001), not ‘accurate’ (*p* = 0.05) or ‘practical’ (*p* = 0.08). The statement with the lowest agreement was ‘For veterinarians, the output provided by ChatGPT is accurate’ (29.7%). Ranked Welch's *t*‐test found that DVM2 students had a higher agreement score for ‘For veterinary students, the output of ChatGPT is practical’ than other cohorts (median = 4 vs. 3, *p* = 0.03), but the difference was not significant within the ANOVA model (*p* = 0.08).

### Perceptions about the impact of AI and ChatGPT in veterinary medicine

**FIGURE 3:**
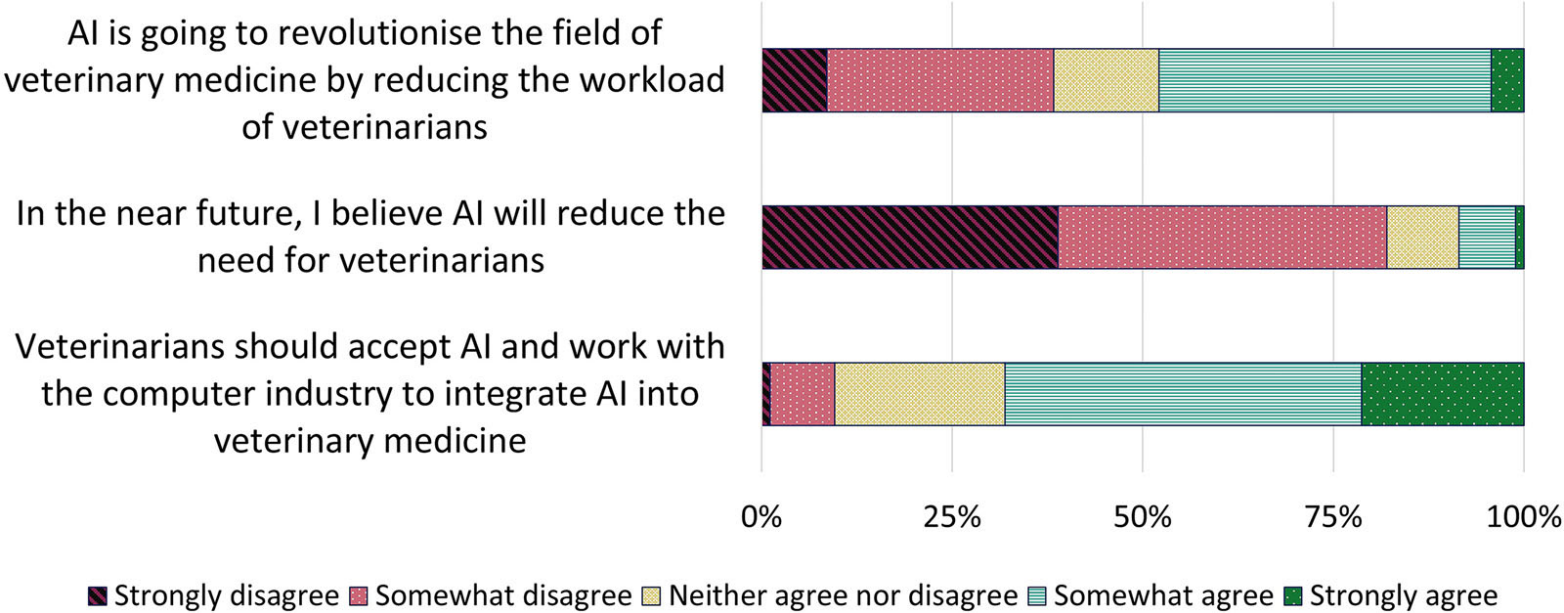
Doctor of Veterinary Medicine (DVM) students’ perceptions about the impact of artificial intelligence (AI) on veterinary medicine. *N* = 95 DVM students from years 1 to 4 at the Sydney School of Veterinary Science.

Responses about the impact of AI did not differ across cohort years, so responses across cohorts were combined (Figure 3). The statement with the highest agreement was ‘Veterinarians should accept AI and work with the computer industry to integrate AI into veterinary medicine’ (68.1%). The statement with the lowest agreement was ‘In the near future, I believe AI will reduce the need for veterinarians’ (8.5%). Just over half (50.5%) of respondents agreed with the statement ‘Basic information about AI and LLMs such as ChatGPT should be included in the veterinary curriculum’ and just under half (47.9%) agreed that ‘AI is going to revolutionise the field of veterinary medicine by reducing the workload of veterinarians’.

Ranked Welch's *t*‐test showed that DVM2 students tended to have stronger agreement with the statement ‘Basic information about AI and LLMs such as ChatGPT should be included in the veterinary curriculum’ than other cohorts (median = 4 in DVM2, median = 3 other cohorts, *p* = 0.03) but the difference was not significant according to the ANOVA model (*p* = 0.08). Across all cohorts, students who responded that they had used tools such as ChatGPT before had higher agreement with the statement ‘Basic information about AI and LLMs such as ChatGPT should be included in the veterinary curriculum’ than those who had heard of ChatGPT but not used it (median = 4 vs. 3, *p* = 0.003).

### Perceptions about ChatGPT after a critical analysis assessment task

**FIGURE 4:**
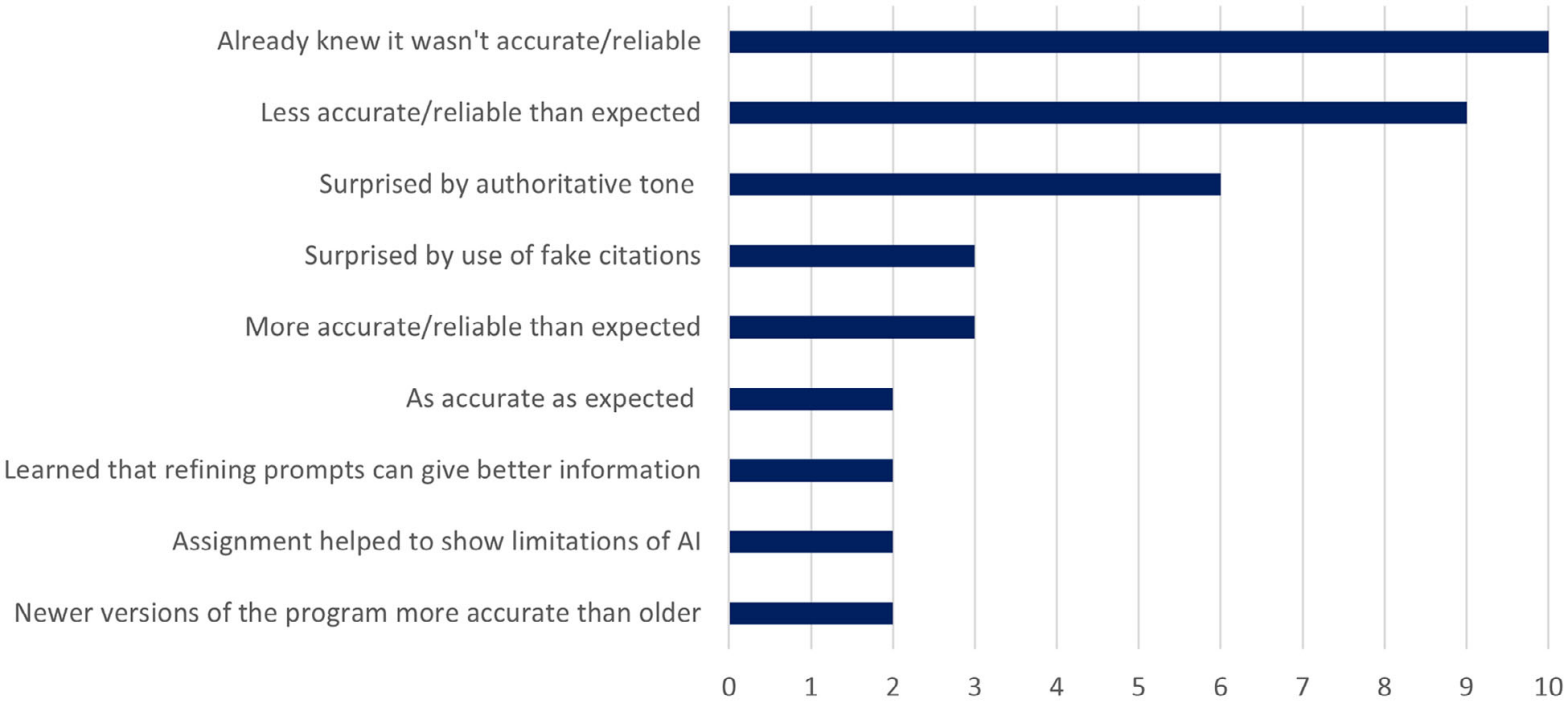
Second‐year Doctor of Veterinary Medicine students’ perceptions about output from artificial intelligence (AI) models such as Chat Generative Pre‐trained Transformer (ChatGPT) after a critical analysis assignment, based on content analysis of free‐text responses to ‘Please comment on why the visual learning tool assignment did or did not change your perception on the accuracy of AI tools such as ChatGPT’ (*N* = 34).

Twenty‐two of 37 (60%) DVM2 students agreed with the statement ‘The VLT assignment that I recently completed changed my perception of the accuracy of AI tools such as ChatGPT’. Figure 4 shows a summary of the free‐text responses given by 34 DVM2 students. In their free‐text responses, 10 of 34 students (29%) said they already knew that ChatGPT was not always accurate. One student commented as follows:

1. ‘It didn't really change my perception of it. AI at its current level of sophistication is about as useful as a google search to anyone working in highly specific fields like medicine … I would personally prefer that kind of bot be used for more menial tasks like summarizing a paragraph … or putting something technical into layman's terms’.

Nine of 34 students (26%) said ChatGPT was less accurate than they expected. One student described their thoughts as follows:

2. ‘I have heard of ChatGPT before … as a tool that many students use to write “perfect” essays. After using ChatGPT for the first time as part of the VLT (assignment), I have found that it only touched on general features of my chosen pathogen …. Oftentimes, it also incorporated features of the human (pathogens) … which was not proven by literature’.

Several students (6/34, 18%) specifically mentioned that they were surprised at the authoritative tone of the ChatGPT output.

3. ‘It was alarming to see the AI model can present information with such authority, even if it is wrong. It is worrying to think about how people without medical training and expertise in the subject matter will accept such information as truth, and the consequences this may have for the health of people's pets/livestock’.

## DISCUSSION

Over 75% of DVM student respondents reported that they were already using AI tools such as ChatGPT. Students strongly believed that AI will not reduce the need for veterinarians in the future. Students who had undertaken critical analysis of ChatGPT perceived ChatGPT output to be more practical than those who had not and believed more strongly that information about ChatGPT should be included in the veterinary curriculum. A pre‐ and post‐survey, conducted after a similar assignment for secondary school physics students, found that after completing the assignment, students agreed more strongly that learning how to use ChatGPT was important in their education.^19^

Despite less than half of the respondents perceiving ChatGPT output to be accurate, more than half agreed that ChatGPT output was relevant and practical for veterinary students, indicating that they perceive this technology to be useful and easy to use, respectively.^12^, ^13^ The majority of respondents indicated that they were already using ChatGPT, which fits with the TAM framework assertions that perceived usefulness and ease of use drive a behavioural intention to use technology.^10^ The fact that DVM2 students, who had been exposed to the critical analysis task, found ChatGPT to be more practical than their peers suggested that the critical analysis assignment may have improved student confidence in navigating ChatGPT.

The disparity between students generally perceiving ChatGPT output as relevant and practical for veterinary students, but not accurate, perhaps points to their view that ChatGPT is a useful tool for summarising background information, although one that cannot be relied on to provide accurate information about veterinary medicine. Similarly, another study^20^ found that the main concern of computer engineering students was that ChatGPT was not accurate enough to help them with assignments and that a high level of background knowledge would be required for them to determine accuracy. In the current study, the fact that the students rated ChatGPT output as more accurate, relevant and practical for veterinary students than pet owners may reflect a belief that they see themselves as an expert compared to pet owners. This was reflected in free‐text comments. Similarly, another study^21^ found that first‐ and second‐year physics students could more easily recognise inaccuracies in ChatGPT output if the content was simple or well known to them; students could not correctly evaluate ChatGPT responses for difficult concepts that were unfamiliar to them.

The perception that ChatGPT output is less accurate for veterinarians than for students could be reflective of the perception that the level of detail in ChatGPT is inadequate for veterinarians but sufficient for students. Students perceiving ChatGPT to be more useful for themselves than pet owners or veterinarians could also reflect a feeling of ‘safety’ when students tackle hypothetical situations in a supportive educational environment rather than dealing with real‐life consequences that could eventuate if a pet owner or veterinarians acts on inaccurate information from ChatGPT. Some DVM2 students who completed the critical analysis assignment noted that they could elicit more accurate answers if they refined the prompts given to ChatGPT. This observation echoes a study^22^ that reported that the accuracy of ChatGPT diagnoses for human medical conditions was governed by the questions asked by the prompter.

Students (DVM2) who had critically evaluated ChatGPT output often noted that they were surprised by its authoritative tone and the inclusion of fake references to support assertions made by ChatGPT. One study^4^ found that ChatGPT provided plausible‐sounding, yet completely falsified references when pressed. In contrast, another study^23^ reported that ChatGPT can provide accurate textbook references when asked to answer questions about medical microbiology. The accuracy of ChatGPT output about veterinary medicine can reasonably be expected to lag behind that of human medicine because of the much larger body of literature about human health compared to that on animal health.

Most respondents agreed that veterinarians should accept the use of AI. Meanwhile, only 8.5% of students agreed that AI would replace the need for veterinarians in the near future. This is similar to a survey of Spanish medicine students studying radiology.^24^ The perception that veterinarians will not be replaced by AI is likely multifactorial, reflecting students’ knowledge of the requirement for a valid veterinarian‒client‒patient relationship,^25^ the notion that AI is not capable of complex tasks such as clinical decision making^26^ and the fact that AI cannot perform hands‐on tasks such as surgery or physical examination.^27^ Less than 50% of students agreed that AI could reduce the workload of veterinarians, perhaps indicating a lack of awareness about the tasks in veterinary medicine where AI has indeed already demonstrated proficiency, such as interpreting radiographs^2^ and prognosticating equine colic cases.^3^

### Implications for veterinary education and clinical practice

Our findings suggest that an explicit task requiring students to critically evaluate ChatGPT output is more effective at highlighting the strengths and weaknesses of AI and LLM than simply providing students with reading material or guidelines about the potential pitfalls of ChatGPT. We recommend that any critical analysis assignment involves a preliminary element where students research a topic to allow them to become ‘experts’ and then an appraisal element where students then apply their expert knowledge to better assess the accuracy of ChatGPT output. We concede that while the critical analysis assignment was useful in highlighting some strengths and weaknesses of ChatGPT for students, future similar assessments should include scope for students to assess the influence of different prompts on the quality of ChatGPT responses. We recommend that issues about ChatGPT language and tone are highlighted in critical analysis assignments so that students can reflect upon the effect of authoritative tone and seeming accuracy of the output. Given students’ disagreement with the statement that AI could reduce the workload of veterinarians in the future, the veterinary curriculum about AI should include information about the tasks where it is already being used in veterinary medicine, or where it could reasonably be expected to be implemented in the future.

This study was limited by the population of students being situated in one institution. A multicentre study that compared responses across veterinary schools and countries would better show the overarching perceptions about AI and ChatGPT. There is a chance that words such as ‘relevance’ and ‘practicality’ could have been interpreted differently by students. Having free‐text responses in future studies would allow respondents to elaborate on what they find relevant or practical about ChatGPT output. We did not ask students in this study to specify how they are currently using ChatGPT. Follow‐up studies would benefit from investigating when and where veterinary students are using the tool and in what circumstances they find it most helpful.

Over 75% of surveyed veterinary students reported already using ChatGPT. This warrants serious attention in veterinary curricular design. Veterinary medical educators should work with students on how to best integrate ChatGPT into their studies. Students who had used ChatGPT perceived it to be relevant and practical, although inaccurate. Less than half of the respondents thought that AI would reduce the workload of veterinarians, suggesting a gap in their understanding about the potential future impacts of AI on veterinary medicine. Veterinary students reported that a critical analysis task changed their perceptions about ChatGPT, indicating that students who can critically engage with AI and LLMs are likely to be better equipped to engage with these tools when they inevitably encounter them in their future careers as veterinarians.

## AUTHOR CONTRIBUTIONS

Kate A. Worthing and Jan Šlapeta designed and delivered the assessments for DVM2 students. Kate A. Worthing, Madeleine Roberts and Jan Šlapeta conceptualised the survey. Jan Šlapeta built the survey. Kate A. Worthing analysed the survey data and wrote the initial manuscript. Madeleine Roberts and Jan Šlapeta reviewed and refined the final manuscript.

## CONFLICTS OF INTEREST STATEMENT

The authors declare they have no conflicts of interest.

### ETHICS STATEMENT

The authors confirm that the ethical policies of the journal, as noted on the journal's author guidelines page, have been adhered to. Approval for the survey was obtained through the Human Ethics Committee at The University of Sydney (2023/346). All participation was with informed consent.

## Supporting information

Supporting Information

## Data Availability

The survey described in this study is available in Appendix S2.
