# Supplementary material for: Surveyed veterinary students in Australia find ChatGPT practical and relevant while expressing no concern about artificial intelligence replacing veterinarians
Source: Vet Rec Open. 2024 Jun 9;11(1):e280. doi: 10.1002/vro2.80 (PMC11162838; doi:10.1002/vro2.80)
Supplement: Supplementary file 1 — Supporting Information [file VRO2-11-e280-s001.pdf]

Supporting Information

Appendix S1 Description of items used in a survey about ChatGPT of Doctor of Veterinary Medicine students at The University of Sydney, Australia

Categorical items

- Year of enrolment in the DVM (1, 2, 3 or 4). No other demographic data was collected.
- Previous experience with ChatGPT (Never used it, heard of it but never used it, used). The survey was set to end at this question for any respondents who had not heard of ChatGPT.
- Whether ChatGPT had been discussed in their curriculum (yes or no). The survey forked so that only respondents who responded 'yes' to this question were asked to answer TAM items or curriculum Likert-like items. For second year DVM students, the following statement: 'The Visual Learning Tool assignment that I recently completed changed my perception of the accuracy of AI tools such as ChatGPT' (true, false).

Likert-style items

Participants were given a set of statements and Likert-style scales to measure the extent to which they agreed with each statement (1= strongly disagree, 2= somewhat disagree, 3= neither agree nor disagree, 4= somewhat agree, 5= strongly agree). Likert-style items included:

- A statement matrix based on the Technology Acceptance Model (TAM) with modifications.[11] Statements included: 'For veterinary students, the output of AI tools such as ChatGPT is accurate/relevant/practical'. This question was repeated 'For pet owners/farm animal owners' and 'For veterinarians'.
- Statements about ChatGPT in the curriculum. These included: 'Prior to the discussion about ChatGPT in my veterinary education, I was already familiar with the strengths and weaknesses of artificial intelligence (AI) tools such as ChatGPT.' The question was repeated for 'Following discussion about ChatGPT in my veterinary education…'
- Statements about the impact of AI and LLMs on veterinary medicine, modified from Galán et al.[24] These included: 'In the near future, I believe AI will reduce the need for veterinarians.', 'AI is going to revolutionise the field of veterinary medicine by reducing the workload of veterinarians', 'Veterinarians should accept AI and work with the computer industry to integrate AI into veterinary medicine' and 'Basic information about AI and large language models (LLM) such as ChatGPT should be included in the veterinary curriculum'. All participants were asked this question.

Free text questions

- For DVM2 students: 'Please comment on why the Visual Learning Tool assignment did or did not change your perception on the accuracy of AI tools such as ChatGPT.'

Appendix S2 Summary of the survey questions about ChatGPT

Thank you for considering participation in our survey on veterinary students' perceptions of the usefulness of artificial intelligence in veterinary medicine.

As veterinary students, some of you have been exposed to ChatGPT – an AI tool also known as a large language model (LLM). You may have experienced its capacity to respond to prompts revolving around pathogenesis of an infectious agent, or the generation of a differential diagnosis for a patient. We now ask you to consider and reflect on such experience and answer few questions.

By clicking 'submit' at the end of the survey, you will be consenting to participate in a research study examining veterinary students' perceptions about the use of artificial intelligence in veterinary medicine. Your participation is completely voluntary, and there is no penalty for choosing not to participate.

The survey should take no more than 10 minutes to complete. All of your responses will remain anonymous and confidential. We will not collect any identifying information about you, such as your name or student ID number. Your responses will be used for research purposes and to improve the DVM curriculum, and no personally identifiable information will be shared with any third parties.

The data we collect will be used to help us better understand veterinary students' attitudes and perceptions about the use of artificial intelligence in veterinary medicine. We hope that the results of our study will contribute to the advancement of veterinary medicine and education.

If you have any questions or concerns about the survey or the research study, please do not hesitate to contact us at emailaddressa@sydney.edu.au or emailaddressb@sydney.edu.au.

Thank you for your participation.

Survey questions

1. What year of the DVM degree are you enrolled in?
    - DVM 1
    - DVM 2
    - DVM 3
    - DVM 4

2. What is your prior experience with ChatGPT? (If the answer is ‘I’ve never heard of ChatGPT’, the survey stops here).
    - I’ve never heard of ChatGPT
    - I’ve heard of ChatGPT but never used it
    - I’ve used ChatGPT

3. Has ChatGPT been discussed in the veterinary education you have received?
    - Yes
    - No

4. (If yes for question 3) To what extent do you agree with this statement: Prior to the discussion about ChatGPT in my veterinary education, I was already familiar with the strengths and weaknesses of artificial intelligence (AI) tools such as ChatGPT.
    - Strongly disagree
    - Somewhat disagree
    - Neither agree nor disagree
    - Somewhat agree
    - Strongly agree

5. (If yes for 3) To what extent do you agree with this statement: Following the discussion about ChatGPT in my veterinary education, I was more familiar with the strengths and weaknesses of artificial intelligence (AI) tools such as ChatGPT.
    - Strongly disagree
    - Somewhat disagree
    - Neither agree nor disagree
    - Somewhat agree
    - Strongly agree

To what extent do you agree with the following statements?

6. For veterinary students, the output provided by AI tools such as ChatGPT is:

	Strongly disagree	Somewhat disagree	Neither agree nor disagree	Somewhat agree	Strongly agree
Accurate	○	○	○	○	○
Relevant	○	○	○	○	○
Practical	○	○	○	○	○

7. For pet owners/farm animal managers, the output provided by AI tools such as ChatGPT is:

	Strongly disagree	Somewhat disagree	Neither agree nor disagree	Somewhat agree	Strongly agree
Accurate	○	○	○	○	○
Relevant	○	○	○	○	○
Practical	○	○	○	○	○

8. For veterinarians, the output provided by AI tools such as ChatGPT is:

	Strongly disagree	Somewhat disagree	Neither agree nor disagree	Somewhat agree	Strongly agree
Accurate	○	○	○	○	○
Relevant	○	○	○	○	○
Practical	○	○	○	○	○

9. In the near future, I believe AI will reduce the need for veterinarians:
    - Strongly disagree
    - Somewhat disagree
    - Neither agree nor disagree
    - Somewhat agree
    - Strongly agree

10. AI is going to revolutionise the field of veterinary medicine by reducing the workload of veterinarians:
    - Strongly disagree
    - Somewhat disagree
    - Neither agree nor disagree
    - Somewhat agree
    - Strongly agree

11. Veterinarians should accept AI and work with the computer industry to integrate AI into veterinary medicine
    - Strongly disagree
    - Somewhat disagree
    - Neither agree nor disagree
    - Somewhat agree
    - Strongly agree

12. Basic information about AI and large language models (LLM) such as ChatGPT should be included in the veterinary curriculum
    - Strongly disagree
    - Somewhat disagree
    - Neither agree nor disagree
    - Somewhat agree
    - Strongly agree

13. (For 2nd year DVM students only) The Visual Learning Tool assignment that I recently completed changed my perception of the accuracy of AI tools such as ChatGPT
    - True
    - False

14. (If true for question 13) Please comment on how the Visual Learning Tool assignment changed your perception on the accuracy of AI tools such as ChatGPT.

15. (If false for question 13) Please comment on why the Visual Learning Tool assignment did not change your perception on the accuracy of AI tools such as ChatGPT.
